# Aspects épidémiologiques des traumatismes du rachis: à propos de 139 cas

**DOI:** 10.11604/pamj.2017.26.16.11350

**Published:** 2017-01-16

**Authors:** Joseph Synèse Bemora, Willy Francis Rakotondraibe, Mijoro Ramarokoto, Willy Ratovondrainy, Clément Andriamamonjy

**Affiliations:** 1 Service de Neurochirurgie, Centre Hospitalier Universitaire Joseph Ravoahangy Andrianavalona Université d’Antananarivo, Madagascar; 2 Service de Neurochirurgie, Centre Hospitalier de Soavinandriana, Université d’Antananarivo, Madagascar

**Keywords:** Fall, head injury, multiple trauma, spine injury, Fall, head trauma, spinal trauma, polytrauma

## Abstract

Les traumatismes du rachis représentent une des lésions les plus fréquemment observées, chez les victimes d’accidents de circulation, d’accident de sportifs, domestique et du travail. Il s’agissait d’une étude rétrospective de 3 ans portant sur 139 cas de traumatismes du rachis hospitalisés et pris en charge dans le service de neurochirurgie du CHUJRA Madagascar. A travers cette étude, 25,17% des traumatisés étaient entre 21 et 30 ans avec une nette prédominance masculine de 69,78% (sexe ratio 2,3). L’étiologie était dominéepar la chute dans 33,09% des cas avec des facteurs de risque dont prise d’alcool (8,63%). Le traumatisme entrait dans le cadre d’un polytraumatisme dont un traumatisme crânien dans 34,63%. Les patients étaient admis dans le service 1 à 5h après le traumatisme dans 31,65% en utilisant comme moyen de transport une voiture personnelle dans 36,69%. Pendant l’hospitalisation 20 patients ont signé une décharge et 6,34% des patients sont décédés. Les traumatismes du rachis posent un problème de santé publique avec une prise en charge lourde surtout pour les patients déficitaires à vie. Devant tout traumatisme du rachis, il faut rechercher systématiquement une lésion crânienne.

## Abstract (en)

Spinal trauma is one of the most common types of injuries among victims of traffic accidents, sports accidents, domestic accidents and workplace accidents. We conducted a 3-year retrospective study of 139 cases of spinal trauma hospitalized and treated in the Neurosurgery department of the CHUJRA, Madagascar. This study shows that 25.17% of injured patients were between 21 and 30 years of age, with a clear male predominance (69.78%; sex ratio 2.3). Falls were the dominating traumatic injury mechanism (33.09%) with risk factors including alcohol use (8.63%). Spinal injuries occurred in patients with polytrauma, of whom 34.63% had cranial trauma. Patients were admitted to the department within 1-5 hours after the trauma in 31.65% of cases, using private car as their means of transport 36.69% of cases. During the hospitalization 20 patients signed the discharge form and 6.34% of patients died. Spinal trauma is a public health problem requiring high intensity management, especially for patients with life-long disabilities. Any spine trauma requires a diligent search for cranial lesion.

## Introduction

La plupart des traumatismes rachidiens sontpris en charge par les centres hospitaliers universitaires, car ilstouchent souvent des patients polytraumatisés. Les traumatismes du rachis sont des lésions sévères pouvant compromettre le pronostic fonctionnel et parfois vital des blessés [1]. L’incidence mondiale est estimée entre 15 et 40 nouveaux cas par million d’habitants. Ce qui représente en France environ 2000 personnes touchées chaque année; 236 nouveaux cas par million d’habitant en Inde et 1800 nouveaux cas par million d’habitant aux Etats-Unis d’Amérique [2, 3]. A Madagascar, il y a peu de données concernant la traumatologie rachidienne c’est la raison pour laquelle nous avons fixé comme objectif de déterminer la fréquence et les aspects épidémiologiques des traumatisés du rachis pris en charge et hospitalisé au service de neurochirurgie du Centre Hospitalier Universitaire Antananarivo de l’Hôpital Universitaire Joseph Ravoahangy Andrianavalona (CHUA-HUJRA) Madagascar.

## Méthodes

Il s’agissait d’une étude rétrospective monocentrique sur des patients hospitalisés et pris en charge dans le service de neurochirurgie du CHUA-HUJRA pendant une période de 3 ans allant du Juillet 2012 à Juillet 2015. Notre recherche est focalisée sur les dossiers complets de tous les patients hospitalisés, les dossiers incomplets ont été exclus. Les paramètres étudiés étaient: l’âge et le sexe des patients, l’étiologie et les facteurs de risque des traumatismes, les moyens de transport des blessés et la durée entre l’accident et l’arrivée à l’hôpital.

## Résultats

A travers cette étude nous avons colligé 139 cas de patients traumatisés du rachis dont 29,50% étaient un traumatisme cervical et 70,50% thoraco-lombaire. Les traumatismes du rachis ont étéobservés surtout chez les sujets jeunes (Tableau 1) de sexe masculin dans 69,78% avec unsexe ratiode 2,3 et un âge moyen de 28,2. L’étiologie des traumatismes du rachis était variable (Tableau 2). La chute par divers raisons sauf dans le lieu de travail était la plus fréquente (33,09%) suivie par les accidents de travail (25,17%).

**Tableau 1:** Répartition des patients traumatisés selon l’âge

Age	Nombre	Pourcentage (%)
0 à 10	5	3,59
11 à 20	13	9,35
21 à 30	35	25,17
31 à 40	24	17,26
41 à 50	26	18,7
51 à 60	22	15,82
< 60	14	10,07
Total	139	100

Les traumatismes du rachis ont été observés surtout chez les sujets jeunes de sexe masculin dans 69,78 % avec un sexe ratio de 2,3 et un âge moyen de 28,2

**Tableau 2:** Répartition selon les étiologies du traumatisme

Etiologie		Nombre	Pourcentage (%)
Accident de circulation	Automobiliste	25	17,98
	Motocycliste	7	5,03
	Cycliste	2	1,43
	Piétons	10	7,19
Chute		46	33,09
Agression		9	6,47
Arme blanche		2	1,43
Arme à feu		3	2,15
AT		35	25,17
AD		29	20,86
AS		8	5,75

La chute par divers raisons sauf dans le lieu de travail était la plus fréquente (33,09%) suivie par les accidents de travail (25,17 %)

**Figure 1:**
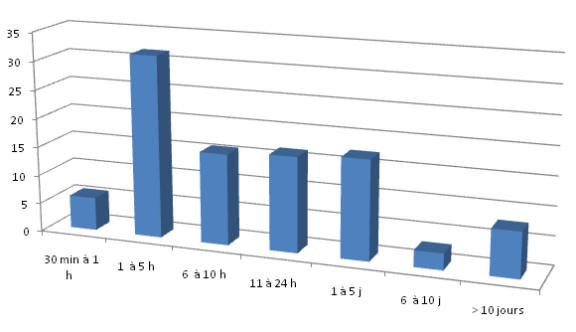
Répartition des patients selon la durée entre traumatisme et la prise en charge hospitalière

Les facteurs de risque associés étaient la prise d’alcool (8,63%) et l’excès de vitesse (3,59%). La majorité des patients ont été référés par les urgentistes dans 83,87%, d’autres ont été référés par un neurochirurgien ou Médecin généraliste avec un taux respectif de 2,15%, consultation directe 1,43%. Les lésions associées étaient un traumatisme crâniens 34,53%, thoracique 12,23%, membres 8,63%, abdominal 3,59% et facial 0,71% des cas. Le délai entre le traumatisme et la prise en charge était variable avec une durée de 1 à 5h dans 31,65% des cas (Figure 1).

Les victimes ont été transportées par une voiture personnelle dans 36,69%; 24,46% par un taxi; 5,75% par une ambulance, par un avion 2,15% et le 30,95% restant par divers moyens. Notre moyen diagnostic était spécialement la radiologie standard simple réalisée dans 85,61% et un scanner dans 23,02%, l’IRM n’était réalisée que dans 0,71% des cas. Pour les patients qui avaient des déficits neurologiques (46 cas), 32,60% ont eu une amélioration complète, 47,82% une amélioration partielle et 19,56% sans amélioration. Pendant l’hospitalisation 7,19% des patients ont signé une décharge. Le taux de mortalité était non négligeable elle tournait aux alentours de 8,63% dont la plupart étaient des traumatismes du rachis cervical (6,47%).

## Discussion

Dans notre étude, la majorité des patients traumatisés du rachis étaient des blessés thoracique et/ou lombaire (70,50%), contrairement à la littérature [4–6]. La prédominance du sujet jeune de sexe masculin (69,78%) est démontrée aussi bien dans notre étude que dans la littérature [2, 7–9]. Dans notre série, l’étiologie est dominé par une chute de divers raison sauf lors du travail dans 39% des cas et suivi par un accident de travail contrairement à ceux dans la littérature qui mentionne que l’accident de la voie public est la première cause [2, 8]. Par faute de moyens, le scanner était l’imagerie par excellence demandé dans 23,02%, ainsi nous n’avons pu poser avec certitude le diagnostic d’une contusion médullaire qui est de diagnostic IRM [10, 11]. Le taux de mortalité était de 8,63% dont la plupart étaient des traumatismes du rachis cervical. Un patient traumatisé du rachis c’est un polytraumatisé dont une atteinte crânienne dans 34,53%, fracture des membres dans 8,63%qu’il faut rechercher de façon systématique chez tous blessés rachidiens. Dans la littérature, Anderson [12] souligne également qu’une fracture des membres se rencontre dans 8% des cas.

Dans la plupart des cas il s’agit d’une pathologie qui met en jeu le pronostic fonctionnel [4] mais elle peut mettre en jeu le pronostic vital (8,63% de décès) dans le cadre d’un traumatisme du rachis cervical (29,50% dans notre série) lié aux troubles respiratoires [13, 14].

## Conclusion

Le traumatisme du rachis est grave et pose un problème de santé publique du fait de la charge imposée par les patients déficitaires surtout les tétraplégiques. L’éducation et la sensibilisation restent primordiales. A Madagascar, un pays en cours de développement, la prise en charge de cette pathologie reste difficile vu le plateau technique inadéquat.

### Etat des connaissances actuelles sur le sujet

- Le traumatisme du rachis touche surtout le sujet jeune de sexe masculin, dont l’étiologie principale est l’accident de la voie public;
- Les lésions sont surtout cervicales dans la majorité des cas.

### Contribution de notre étude à la connaissance

- Point de vu différente sur l’étiologie des traumatismes du rachis: chute;
- Situation actuelle de Madagascar sur le traumatisme du rachis.
